# *Pinus pinea* L. Periderm as a Sustainable Source of Phenolic Bioactives: Integrated Phytochemical, Antioxidant, and Elemental Investigation

**DOI:** 10.1002/cbdv.71561

**Published:** 2026-08-08

**Authors:** Vlad Sebastian Popescu, Fabjola Bilo, Eileen Mac Sweneey, Laura Borgese, Gregorio Peron, Giulia Abate, Andrea Mastinu

**Affiliations:** ^1^ Department of Molecular and Translational Medicine Division of Pharmacology University of Brescia Brescia Italy; ^2^ Department of Medical and Surgical Specialties Radiological Sciences and Public Health University of Brescia Brescia Italy; ^3^ Department of Mechanical and Industrial Engineering University of Brescia Brescia Italy

**Keywords:** antioxidant activity, periderm, *Pinus pinea*, taxifolin, TXRF, UPLC–QTOF–MS

## Abstract

*Pinus pinea* L. is a widespread Mediterranean conifer whose bark‐derived tissues remain poorly investigated as potential sources of bioactive secondary metabolites. In this study, a hydroalcoholic crude extract of the periderm, an outer renewable bark layer compatible with sustainable collection, was evaluated as an underexplored natural matrix. HPLC–DAD identified taxifolin as the main quantified marker compound, corresponding to 21.1 mg/g of dry extract. Untargeted UPLC–QTOF–MS analysis revealed a polyphenol‐rich profile, mainly represented by condensed tannins and flavonoids in ESI(−) mode, while terpenoids were more evident in ESI(+) mode. The extract showed concentration‐dependent DPPH radical‐scavenging activity, with an IC_50_ of 12.31 µg/mL, comparable to ascorbic acid. Comparison with taxifolin tested at concentrations equivalent to those estimated in the extract indicated that taxifolin accounted for only part of the observed antioxidant response. In SH‐SY5Y cells, the extract was cytocompatible up to 1 µg/mL and partially counteracted H_2_O_2_‐induced reduction in cell viability within this non‐cytotoxic range. TXRF analysis provided a preliminary safety‐oriented elemental profile, with regulated elements detected at low levels. Overall, these findings support *P. pinea* periderm as a renewable Mediterranean source of secondary metabolites with antioxidant potential, while further mechanistic and toxicological studies are required to confirm its functional applicability.

## Graphical abstract

A hydroalcoholic extract from the renewable periderm of *Pinus pinea* is characterized through integrated phytochemical, biological and elemental analyses. The extract exhibits a phenolic‐rich composition, significant antioxidant activity and preliminary protection against oxidative stress in SH‐SY5Y cells, while elemental profiling indicates low levels of regulated metals, supporting its potential as a sustainable source of bioactive compounds.

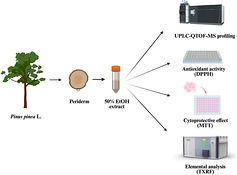

## 1 Introduction

The stone pine *Pinus pinea* L. is a long‐lived conifer characteristic of coastal and inland Mediterranean landscapes, extending from the Iberian Peninsula to the Eastern Mediterranean [1]. Besides its ecological role in stabilizing sandy soils, shaping dune ecosystems, and contributing to fire‐resilient forests, *P. pinea* also has considerable economic and cultural value [2]. Its edible seeds, commonly known as pine nuts, are among the most valuable non‐wood forest products in the Mediterranean region [3]. Several species of the genus *Pinus* have also been investigated as sources of biologically active secondary metabolites. Pine resins, oleoresins, bark, and needle preparations have a documented history of traditional use in Europe and the Mediterranean area, particularly for respiratory, inflammatory, and skin‐related conditions [4, 5, 6, 7, 8]. Most reports concern species such as *Pinus pinaster*, *Pinus sylvestris*, and *Pinus halepensis* [9], whereas *P. pinea* has received comparatively less attention. Nevertheless, available ethnobotanical syntheses indicate that *P. pinea* is also among the European pine taxa with reported traditional applications, including resin‐ and bark‐derived preparations for topical and respiratory uses [4].

Despite this long‐standing traditional relevance, and despite the economic importance of pine nuts, *P. pinea* has received remarkably little phytochemical investigation compared to other conifer species. This is particularly striking given the extensive body of literature available for related taxa. *P. pinaster* bark is among the best‐studied tree tissues worldwide, known for its high content of procyanidins, phenolic acids and flavonoids, including taxifolin; it is the source of the patented extract Pycnogenol, used for vascular and antioxidant applications [10, 11].

*Pinus gerardiana* seeds and bark contain over 60 identified phytoconstituents, including lignans, flavonoids and terpenes, reflecting its long‐standing use in Himalayan traditional medicine [12]. *P. sylvestris* bark has been shown to contain catechin, epicatechin, myricetin, quercetin, vanillic acid and a variety of stilbenes and tannins, with documented antioxidant, antimicrobial and anti‐inflammatory activities [13]. Other well‐characterized species, such as *Pinus radiata*, *P. koraiensis*, *P. massoniana*, and *Picea abies*, have contributed substantially to the understanding of polyphenol‐rich conifer bark extracts and their biological effects [14].

In contrast, *P. pinea* remains largely underexplored from a phytochemical and bioactivity perspective. While recent investigations have addressed the chemical composition and biological properties of specific tissues, including needle essential oils and metabolite profiling, nut and nutshell extracts, and needle‐derived compounds [7, 15, 16], bark‐derived tissues have received limited attention. This gap is particularly relevant because *P. pinea* is one of the most characteristic and economically important pine species of the Mediterranean basin, where it is widely present and already integrated into agro‐forestry and pine nut production chains. In this context, lignocellulosic residues are generated during tree maintenance, pruning, forestry management, and processing activities. Among these materials, the periderm represents a particularly attractive and neglected matrix. As the outermost suberized bark layer, it is readily accessible, periodically renewed during secondary growth, and compatible with nondestructive collection. Its valorization could therefore contribute to the sustainable upgrading of underused Mediterranean forestry by‐products while providing a renewable source of secondary metabolites. Despite these features, no detailed analytical study has specifically focused on the phytochemical composition and biological properties of *P. pinea* periderm.

Given the rich phytochemical profiles observed in the bark of other pine species, it is plausible that *P. pinea* periderm may also contain bioactive phenolic compounds of therapeutic interest. Conifer bark extracts are particularly recognized for their antioxidant activity [17, 18], which is primarily attributed to polymeric procyanidins, flavonoids such as catechins and taxifolin [19], and various phenolic acids. These compounds are known to modulate oxidative stress pathways, reduce reactive oxygen species (ROS) accumulation, and exhibit cytoprotective, neuroprotective, and anti‐inflammatory activities in several biological models [20, 21, 22].

Oxidative stress, in turn, is strongly implicated in pathological processes and in general cellular dysfunction [23]. The human neuroblastoma cell line SH‐SY5Y is a widely used model for studying ROS‐induced cytotoxicity, neuronal injury, and the protective effects of antioxidants [24, 25]. Numerous phenolics, including taxifolin, catechins, and condensed tannins, have previously been shown to attenuate H_2_O_2_‐induced oxidative damage in this cell line by modulating mitochondrial metabolism, scavenging radicals, or promoting endogenous antioxidant responses [26, 27]. These observations support the use of SH‐SY5Y cells as a suitable in vitro model to preliminarily evaluate the effects of phenolic‐rich extracts under oxidative stress conditions.

In addition to phytochemical and biological characterization, the evaluation of elemental composition is becoming an increasingly important aspect of natural product research. Several studies have demonstrated that botanical supplements, particularly those imported from Asia, may contain elevated concentrations of toxic elements such as lead, cadmium, arsenic, and mercury, often exceeding European regulatory limits [28, 29].

Although heavy metals may not induce immediate toxicity in short‐term cell assays, they can accumulate in the human body after chronic exposure [30]. Therefore, elemental profiling is important for a preliminary safety‐oriented evaluation of plant‐derived extracts intended for potential nutraceutical applications. In this context, the analysis of *P. pinea* periderm and its extract provides complementary information on the presence of regulated and trace elements.

The present study was therefore designed to fill this gap by investigating *P. pinea* periderm through complementary analytical and biological approaches. First, HPLC–DAD and untargeted UPLC–QTOF–MS were used to define the secondary metabolite profile of the crude extract, with particular attention to taxifolin and the main phytochemical classes. The extract was then evaluated for radical‐scavenging activity using the DPPH assay and for its ability to preserve SH‐SY5Y cell viability under H_2_O_2_‐induced oxidative stress. Finally, TXRF analysis was performed to obtain a preliminary elemental profile and to support a preliminary safety‐oriented evaluation of this renewable bark‐derived matrix.

## 2 Materials and Methods

### 2.1 Plant Material and Sampling

Periderm samples of *P. pinea* L. were collected on March 23, 2024, from three mature trees located in Brescia, Italy (Via S. Gottardo; 45.542561°N, 10.252513°E; 350–380 m above sea level). The selected individuals exhibited trunk circumferences ranging from 135 to 150 cm. At the time of sampling, ambient conditions were stable (17°C; relative humidity 61%).

Immediately after collection, the outer bark (periderm) fragments were transported to the laboratory, where they were rinsed with distilled water to remove adhering debris and epiphytic material. Samples were air‐dried at room temperature and subsequently oven‐dried for 48 h until constant weight. The dried material was then finely ground and stored in airtight containers at room temperature until extraction. The plant material was identified by Professor Andrea Mastinu, botanist at the University of Brescia. A voucher specimen (PP‐2024‐01) has been deposited in the reference collection of the Plant Biology Laboratory, Division of Pharmacology, Department of Molecular and Translational Medicine, University of Brescia (Brescia, Italy), where it is permanently retained and available for future reference.

### 2.2 Extract Preparation

The extraction was performed using a hot hydroalcoholic procedure selected based on the optimization study reported by Ferreira‐Santos et al. [10]. Although this method was originally optimized for *P. pinaster* bark, it was considered suitable for the present study because *P. pinaster* and *P. pinea* are closely related conifer species and because the extracted material represented a comparable bark‐derived matrix. In that study, the use of 50% ethanol combined with elevated temperature and extended extraction time provided the highest recovery of phenolic compounds and antioxidant constituents. Therefore, these conditions were adopted as a rational starting point for the extraction of *P. pinea* periderm.

A solid‐to‐liquid ratio of 0.15 g/mL was used by suspending finely ground *P. pinea* periderm in 50% ethanol (v/v). Samples were heated in a thermostatic bath at 82°C for 115 min. After heating, the mixtures were centrifuged at 4000 rpm for 10 min. The resulting supernatant was collected; a portion was stored at 4°C for chromatographic analysis, while the remaining fraction was lyophilized, aliquoted, and stored at −80°C for subsequent biological assays. The extraction yield was calculated as the ratio between the dry weight of the lyophilized extract and the initial dry weight of periderm used for extraction and expressed as a percentage (w/w). Based on dry residue determinations, the hydroalcoholic extraction afforded a yield of 21.5 ± 2.3% (w/w, *n* = 10).

### 2.3 Phytochemical Analysis

#### 2.3.1 HPLC–DAD Analysis

Chromatographic analyses were carried out on a Shimadzu Prominence‐i LC‐2030C system equipped with a binary high‐pressure pump, thermostatic autosampler, and DAD detector.

The chromatographic separation was achieved on a Kinetex Evo C18 column (250 × 4.6 mm, 5 µm, 100 Å) using a mobile phase of 0.1% TFA in water (A) and 0.1% TFA in acetonitrile (B). The gradient started at 5% B for 1 min, then increased linearly to 30% B over 8 min, was held for 1 min, and subsequently ramped to 100% B within 5 min. After a 1‐min hold at 100% B, the system was returned to initial conditions (5% B) and re‐equilibrated for the final minute. The flow rate was 1.0 mL/min, column temperature 40°C, injection volume 10 µL, and detection wavelength 287 nm, for a total run time of 18 min.

The main compound, taxifolin, was quantified using a calibration curve prepared with standard taxifolin ranging from 31.25 to 1000 µg/mL. Results were expressed as µg of compound per mL of extract.

#### 2.3.2 UPLC–QTOF–MS Analysis

Samples were prepared by dissolving 10 mg of dry extract in 1.2 mL of ethanol/water (20:80, v/v). Mixtures were sonicated for 15 min, centrifuged at 13 000 rpm for 10 min, and transferred in a polypropylene vial for analysis.

The UPLC–QTOF–MS method was adapted from a previously published protocol using the same chromatographic and mass spectrometric platform, with minor modifications to sample preparation according to the specific solubility and composition of the *P. pinea* periderm extract [31].

Analyses were performed on a Waters Acquity UPLC system coupled to a Xevo G2 QTOF mass spectrometer, operated in both positive and negative electrospray ionization (ESI) modes. Chromatography was performed using a Waters BEH C18 column (2.1 × 100 mm, 1.7 µm) kept at 40°C as the stationary phase, and a mixture of 0.1% formic acid in water (A) and 0.1% formic acid in acetonitrile (B) as the mobile phase. The flow rate was 300 µL/min. The elution gradient was the following: 0–1 min, 98% A; 11 min, 15% A; 16 min, 0% A; 20 min, 0% A; 21 min, 98% A; 24 min, 98% A. The injection volume was 2 µL. Accurate MS data were acquired in the mass range of 50–2000 Da. The sampling cone voltage was adjusted at 40 V, and the source offset was 80 V. The capillary voltage was set to 3.5 KV. Nitrogen was used as nebulizer gas at a flow rate of 800 L/h. The desolvation temperature was 450°C. Mass accuracy and reproducibility were maintained by infusing lock mass (leucine–enkephalin) through Lockspray at a flow rate of 20 µL/min. The *m/z* value of all acquired spectra was automatically corrected during acquisition based on lock mass. Fragmentation data on the eluted metabolites were acquired by MS^e^, setting the collision energy to 30 V.

Chromatographic and MS data of eluted metabolites were extracted from raw chromatograms and MS spectra using the MarkerLynx XS software (Waters). Tentative identification of metabolites was performed by calculating potential molecular formulas from their isotopic patterns in MS, using the Elemental Composition tool (Waters). The formulas with the highest confidence of fitting (>80%) were then used to search for possible candidates in databases such as PubChem (https://pubchem.ncbi.nlm.nih.gov/), Food Metabolome Database (https://foodb.ca/), and KNApSAcK (https://www.knapsackfamily.com/KNApSAcK/), or in the available literature. Identification was confirmed by evaluating the fragmentation patterns in the MSe spectra.

Semi‐quantification of metabolites was performed by comparison with standard compounds representative of flavonoids (rutin), phenolic acids (chlorogenic acid), and terpenes (azadirachtin). For each standard, five‐point calibration curves were generated within the linear dynamic range of the detector by plotting peak area (AUC) versus nominal concentration. Metabolite abundances were estimated by comparing individual AUC values with the corresponding calibration curves of chemically related representative standards and are expressed as mg of rutin equivalents, chlorogenic acid equivalents, or azadirachtin equivalents per g of dry extract. Since authentic standards were not available for all annotated metabolites, these values should be considered semi‐quantitative estimates rather than absolute concentrations.

### 2.4 Elemental Analysis by Total Reflection X‐Ray Fluorescence (TXRF)

The elemental composition of *P. pinea* 50% hydroalcoholic extract was assessed by total reflection x‐ray fluorescence (TXRF). Sample preparation followed an optimized protocol adapted from Dalipi et al. [32] and Bilo et al. [33]. Briefly, dried and powdered periderm samples (10 mg) were suspended in 990 µL of deionized water and homogenized by vortexing. For each suspension, 10 µL of an internal standard (Ga, final concentration 1 mg/L) was added to allow quantitative analysis. After mixing, 10 µL of the suspension were deposited onto a siliconized quartz glass reflector and dried on a hot plate. Extract samples were prepared similarly by suspending the lyophilized extract (amount adjusted to 10 mg equivalent) in deionized water and adding Ga as an internal standard.

TXRF spectra were acquired using a benchtop TXRF spectrometer (HORIZON, GNR srl, Italy) equipped with a Mo x‐ray tube operating at 40 kV and 15 mA, and a 30 mm^2^ silicon drift detector (SDD). Each reflector was irradiated for 600 s of real time. Spectral deconvolution and quantification were performed using the manufacturer's software. Elemental concentrations were expressed as ppm (µg/g dry weight). All samples were analyzed in triplicate, and the results are reported as mean ± standard deviation (SD).

### 2.5 Antioxidant Activity (DPPH Assay)

The antioxidant capacity of *P. pinea* periderm extract was evaluated using the DPPH (2,2‐diphenyl‐1‐picrylhydrazyl) radical‐scavenging assay, adapted from Christodoulou et al. [34]. A 0.1 mM DPPH solution in methanol was prepared fresh and protected from light. In each well, 150 µL of DPPH solution was mixed with 50 µL of sample or reference standard. Plates were incubated for 30 min at room temperature in the dark, and absorbance was measured at 517 nm using a microplate spectrophotometer.

The extract was tested at concentrations expressed as dry lyophilized extract equivalents. Based on the taxifolin content quantified by HPLC–DAD, the selected extract concentrations of 2.5, 5, 25, and 50 µg/mL corresponded to estimated taxifolin contents of 0.05, 0.10, 0.50, and 1.0 µg/mL, respectively. Taxifolin was therefore tested as a pure reference compound at the corresponding concentrations to assess the contribution of the major quantified flavonoid to the overall antioxidant response of the extract. Trolox and ascorbic acid were included as reference antioxidants.

Sample blanks were included to correct for the intrinsic absorbance of the extract at 517 nm. Radical‐scavenging activity was calculated as the percentage decrease in absorbance relative to the DPPH control. Trolox equivalent and ascorbic acid equivalent values were estimated from the linear regression equations of the respective reference standards. IC_50_ values were determined by nonlinear regression analysis of the dose–response curves using GraphPad Prism software (GraphPad Software, San Diego, CA, USA).

### 2.6 Cell Culture and Treatment

The SH‐SY5Y cell line (human neuroblastoma cells, RRID: CVCL_0019) was cultured in cell culture medium composed of DMEM and Ham's F12 medium 1:1, supplemented with 10% fetal bovine serum, 0.5% L‐glutamine, 100 U/mL penicillin and 100 µg/mL streptomycin, at 37°C and 5% CO_2_. Cells were seeded at a density of 2 × 10^4^ cells/well in 96‐well plates 24 h prior to treatment. Cell culture conditions were based on a protocol previously established by our group for the evaluation of antioxidant activity of natural extracts [35]. The lyophilized *P. pinea* periderm hydroalcoholic extract was reconstituted in complete culture medium, centrifuged at 4.000 rpm for 10 min at 4°C, and then used for the treatment. Taxifolin (Sigma‐Aldrich, Germany) was dissolved in 50% ethanol to obtain a 12.5 mg/mL stock and diluted in medium to the desired experimental concentrations.

### 2.7 Cytocompatibility Assay (MTT)

Cytocompatibility was evaluated using the MTT reduction assay, following a protocol previously established by our group for SH‐SY5Y cells [35], with appropriate modifications for the present experimental conditions. SH‐SY5Y cells were exposed for 24 h to increasing concentrations (0.05, 0.1, 0.5, 1, 10, 25, and 50 µg/mL) of *P. pinea* extract or taxifolin. For comparison purposes, the concentrations of the extract were also expressed according to the taxifolin content previously quantified by HPLC–DAD, allowing normalization of extract doses to taxifolin‐equivalent levels. After treatment, 10 µL of MTT solution (5 mg/mL in PBS) was added to each well, and cells were incubated for 3 h at 37°C. The supernatant was then discarded, and the resulting formazan crystals were solubilized in 100 µL of DMSO. Absorbance was measured at 570 nm using a microplate reader. Cell viability was calculated relative to untreated control cells and expressed as a percentage of metabolic activity.

### 2.8 Oxidative Stress Protection Assay

The protective effect of the *P. pinea* extract against oxidative damage was assessed using an H_2_O_2_‐induced stress model, adapted from a protocol previously applied by our group to evaluate the antioxidant potential of natural extracts in SH‐SY5Y cells [35]. SH‐SY5Y cells were pre‐treated for 24 h with non‐cytotoxic concentrations of the extract or taxifolin (0.05, 0.1, 0.5, and 1 µg/mL). Following pre‐treatment, cells were exposed to 100 µM H_2_O_2_ for 2 h to induce oxidative stress. Cell viability was subsequently evaluated using the MTT assay, as reported above. Results were expressed as the percentage of viable cells relative to untreated control cultures.

### 2.9 Statistical Analysis

Results are expressed as mean ± standard error of the mean (SEM) or mean ± standard deviation (SD), as indicated in the corresponding tables and figure legends. Cell‐based experiments were performed in three independent biological experiments, each including three technical replicates for every experimental condition. For analytical assays (DPPH and TXRF), data are reported as mean ± SD from the number of replicate measurements specified in the corresponding Materials and Methods sections. No data transformation or normalization was applied prior to statistical analysis. Data were inspected for consistency, and no experimental outliers were identified or excluded. Normality of residuals and homogeneity of variances were assessed using the Shapiro–Wilk and Brown–Forsythe tests, respectively. The assumptions required for one‐way ANOVA were considered adequately met. Statistical analysis of cell‐based assays was performed using one‐way analysis of variance (ANOVA) followed by Dunnett's multiple comparison test. Differences were considered statistically significant at *p* < 0.05. Dose–response curves and IC_50_ values were obtained by nonlinear regression analysis. All statistical analyses were performed using GraphPad Prism version 8.0.0 (GraphPad Software, San Diego, CA, USA).

## 3 Results

### 3.1 Phytochemical Profile (HPLC–DAD)

**FIGURE 1:**
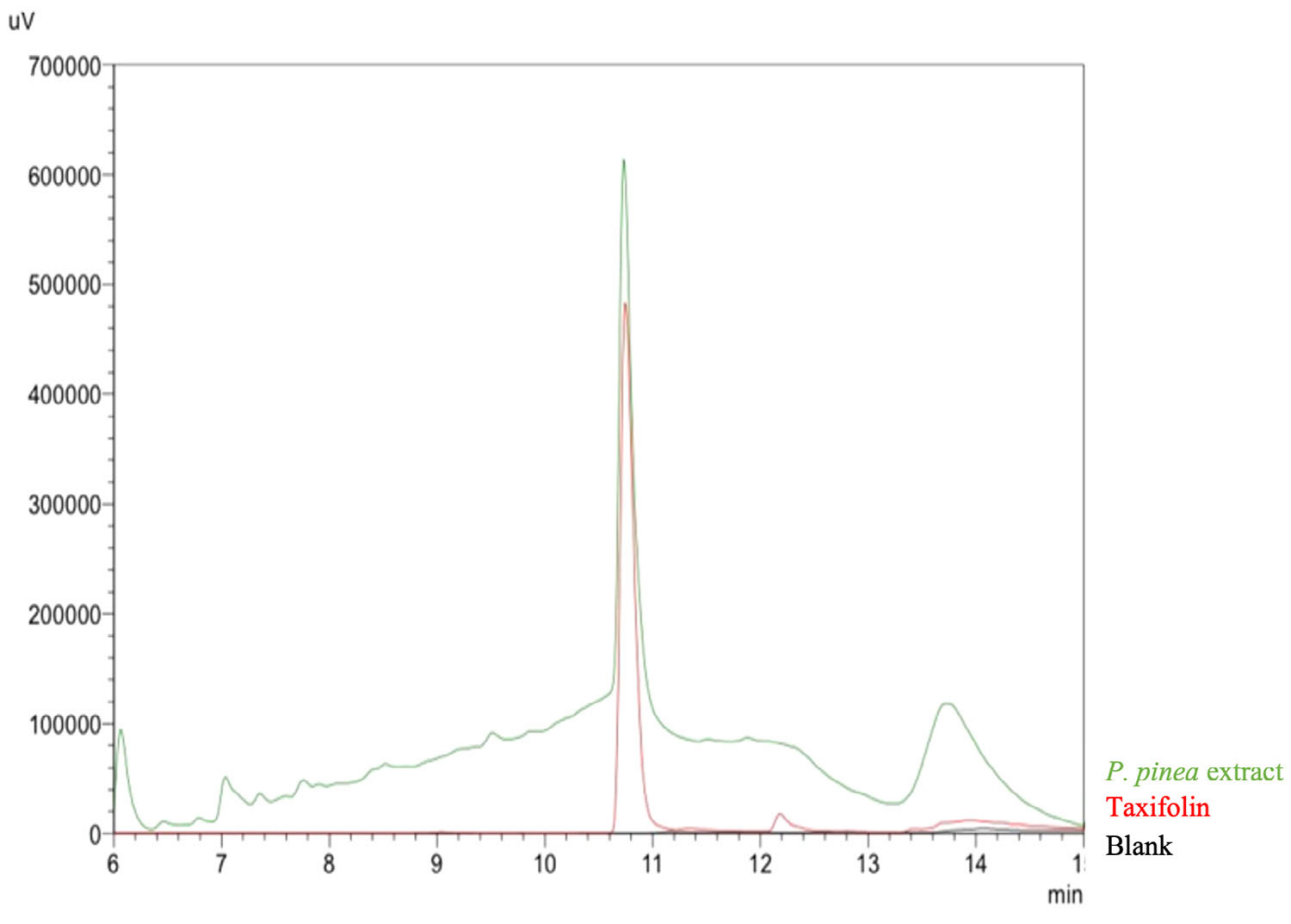
HPLC–DAD chromatographic profiles of *P. pinea* periderm extract and taxifolin standard. Representative HPLC–DAD chromatograms recorded at 287 nm showing the blank (black line), *P. pinea* periderm extract (green line) and taxifolin standard (red line).

HPLC–DAD analysis revealed taxifolin as the predominant phenolic constituent of the *P. pinea* periderm extract (Figure 1). Quantitative determination was performed using an external calibration curve constructed with a commercial taxifolin standard over the range 31.25–1000 µg/mL. The calibration curve showed good linearity (*y* = 43795.9*x*−159217.3; *R* ^2^ = 0.9994), with estimated LOD and LOQ values of 27.6 and 83.5 µg/mL, respectively.

Based on this calibration, the taxifolin concentration in the analyzed extract was 676.01 µg/mL, corresponding to 21.1 mg/g of dry extract. The chromatographic profile monitored at 287 nm was characterized by a dominant taxifolin peak, whereas additional signals attributable to other phenolic constituents were present at lower intensities.

### 3.2 UPLC–QTOF–MS Untargeted Metabolite Profiling

Untargeted UPLC–QTOF–MS analysis enabled the characterization of the secondary metabolite profile of the *P. pinea* periderm crude extract. Metabolites were tentatively annotated based on accurate mass measurements, isotopic patterns, MS/MS fragmentation data, and comparison with reference databases and literature reports. Since authentic reference standards were not available for all detected metabolites, compound identification should be regarded as tentative, except for taxifolin, which was confirmed and quantified using a commercial analytical standard. Overall, the detected metabolites were mainly assigned to the flavonoid, condensed tannin, terpenoid and phenolic acid classes.

**FIGURE 2:**
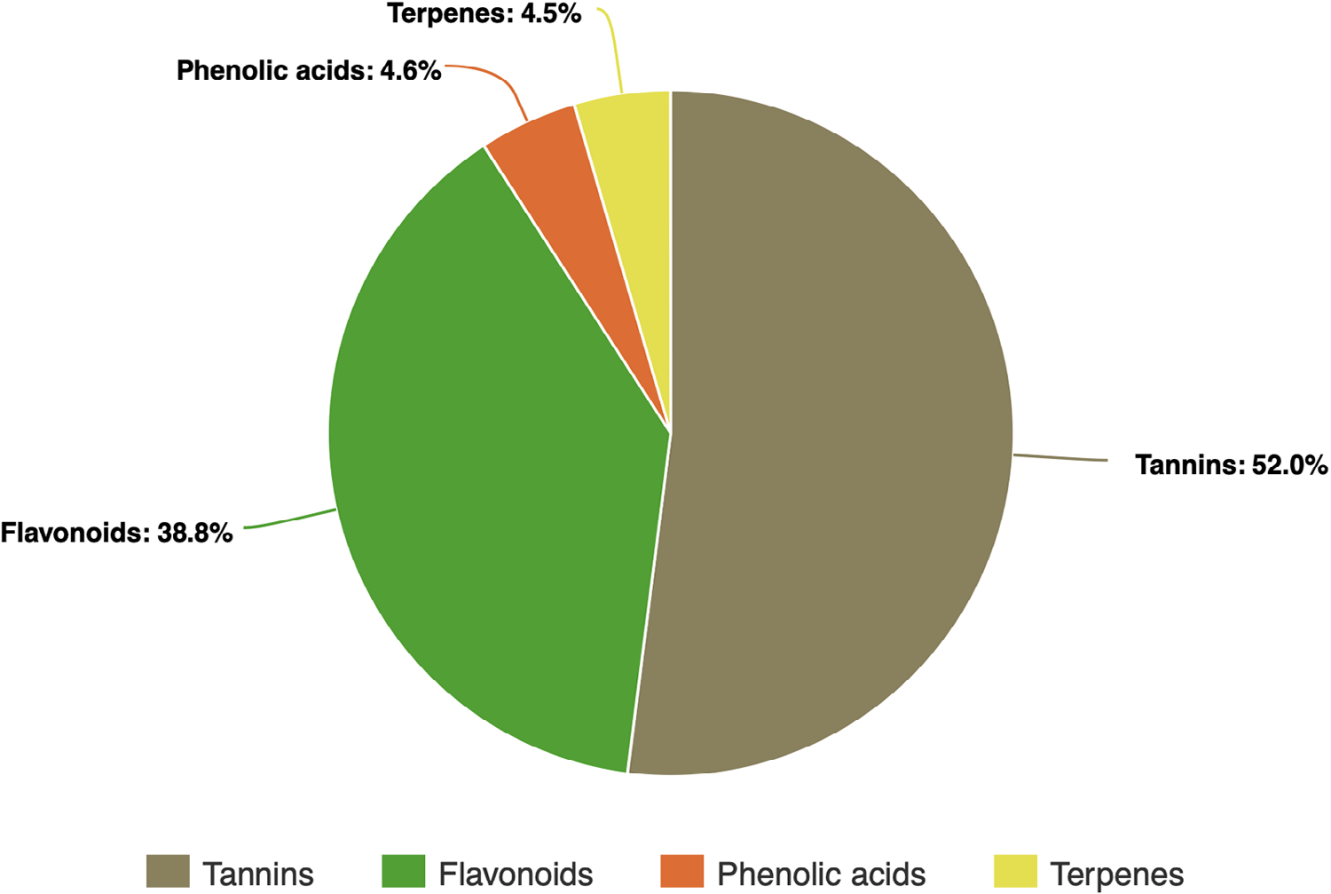
Relative distribution of the phytochemical classes identified in *P. pinea* periderm extract by UPLC–QTOF–MS operating in negative electrospray ionization (ESI−) mode. Percentages represent the relative contribution of each phytochemical class to the total identified compounds, calculated from the quantified amounts expressed as mg/g of dry extract: tannins, 22.19 mg/g; flavonoids, 16.55 mg/g; terpenes, 1.94 mg/g; phenolic acids, 1.97 mg/g.

In negative electrospray ionization mode (ESI−), a total of 42.65 mg/g dry extract of annotated metabolites was quantified (Table 1). As summarized in Figure 2, condensed tannins represented the predominant phytochemical class (22.19 mg/g; 52.0% of the total annotated metabolites), followed by flavonoids (16.55 mg/g; 38.8%), whereas phenolic acids (1.97 mg/g; 4.6%) and terpenes (1.94 mg/g; 4.5%) accounted for smaller proportions. Among the individual metabolites, taxifolin was the most abundant flavonoid (10.94 ± 0.43 mg/g dry extract), followed by prodelphinidin A1 (7.89 ± 0.40 mg/g), kaempferol (2.65 ± 0.22 mg/g), procyanidin B dimer (2.17 ± 0.04 mg/g), and procyanidin C2 isomer 2 (2.15 ± 0.01 mg/g).

**TABLE 1:** Compounds identified in *P. pinea* periderm extract by UPLC–QTOF–MS in ESI(–) mode.

RT (min)	*m/z*	Molecular formula [M–H]^−^	Main fragments	Name	Class	mg/g extract
3.81	289.0716	C_15_H_14_O_6_	245.0811, 125.0233	Epicatechin	Flavonoids	0.28 ± 0.00
3.88	739.1872	C_36_H_36_O_17_	721.1772	Procyanidin B, C‐glucoside isomer 1	Tannins	1.73 ± 0.03
3.97	865.1996	C_45_H_38_O_18_	289.1711	Procyanidin C2 isomer 1	Tannins	0.85 ± 0.04
4.05	577.1349	C_30_H_26_O_12_	425.0865, 407.0763, 289.0709	Procyanidin B dimer	Tannins	2.17 ± 0.04
4.05	451.1237	C_21_H_23_O_11_	289.0709	Catechin 3‐O‐glucoside	Flavonoids	0.40 ± 0.01
4.10	289.0716	C_15_H_14_O_6_	245.0815, 125.0234	Catechin	Flavonoids	1.27 ± 0.02
4.30	865.1994	C_45_H_38_O_18_	289.1712	Procyanidin C2 isomer 2	Tannins	2.15 ± 0.01
4.30	1153.2606	C_60_H_50_O_24_	289.0707	Procyanidin B tetramer	Tannins	1.45 ± 0.02
4.37	863.1815	C_45_H_36_O_18_	125.0231	Procyanidin A trimer isomer 1	Tannins	0.92 ± 0.02
4.50	1441.3206	C_75_H_61_O_30_	289.0709	Procyanidin B pentamer	Tannins	0.41 ± 0.01
4.61	863.1815	C_45_H_36_O_18_	125.0233	Procyanidin A trimer isomer 2	Tannins	0.68 ± 0.02
4.65	865.1996	C_45_H_38_O_18_	289.1712	Procyanidin C2 isomer 3	Tannins	0.57 ± 0.03
4.73	575.1197	C_30_H_24_O_12_	125.0233	Procyanidin A2 isomer 1	Tannins	0.19 ± 0.00
4.73	465.1037	C_21_H_22_O_12_	289.0716	Catechin 4′‐glucuronide	Flavonoids	0.14 ± 0.02
4.82	515.1911	C_27_H_32_O_10_	—	Harrisonin	Terpenes	0.29 ± 0.00
4.83	485.1812	C_26_H_30_O_9_	—	Rutaevin	Terpenes	0.14 ± 0.00
4.85	863.1815	C_45_H_36_O_18_	125.0231	Procyanidin A trimer isomer 3	Tannins	0.47 ± 0.04
4.90	1151.2384	C_60_H_48_O_24_	125.0239	Procyanidin A tetramer	Tannins	1.48 ± 0.05
4.94	575.1197	C_30_H_24_O_12_	125.0231	Procyanidin A2 isomer 2	Tannins	0.61 ± 0.01
4.97	465.1037	C_21_H_22_O_12_	289.0708, 125.0234	Epicatechin 4′‐glucuronide	Flavonoids	0.20 ± 0.00
4.97	449.1088	C_21_H_22_O_11_	303.0507	Taxifolin 3‐rhamnoside	Flavonoids	0.12 ± 0.02
5.10	575.1197	C_30_H_24_O_12_	125.0231	Procyanidin A2 isomer 3	Tannins	0.13 ± 0.00
5.12	303.0509	C_15_H_12_O_7_	285.0401, 125.0233	Taxifolin	Flavonoids	10.94 ± 0.43
5.14	607.1099	C_30_H_24_O_14_	125.0233	Prodelphinidin A1	Tannins	7.89 ± 0.40
5.24	285.0398	C_15_H_10_O_6_	255.0522, 227.0355	Kaempferol	Flavonoids	2.65 ± 0.22
5.36	423.0719	C_22_H_16_O_9_	—	Hypnum acid	Flavonoids	0.23 ± 0.00
5.78	589.0981	C_30_H_22_O_13_	—	Nonahydroxy‐3′,6″‐biflavonone	Tannins	0.35 ± 0.04
6.00	359.1127	C_19_H_20_O_7_	200.9122	Barbatinic acid	Phenolic acids	1.97 ± 0.12
6.05	301.0346	C_15_H_10_O_7_	178.9981, 151.0027	Quercetin	Flavonoids	0.23 ± 0.00
6.05	287.0554	C_15_H_12_O_6_	177.0588, 125.0266	Dihydrokaempferol	Flavonoids	0.09 ± 0.02
7.28	821.3962	C_42_H_62_O_16_	747.3961	Uralsaponin B	Terpenes	1.30 ± 0.01
7.57	807.4177	C_42_H_64_O_15_	—	Licoricesaponin B2 analogue	Terpenes	0.21 ± 0.04

**FIGURE 3:**
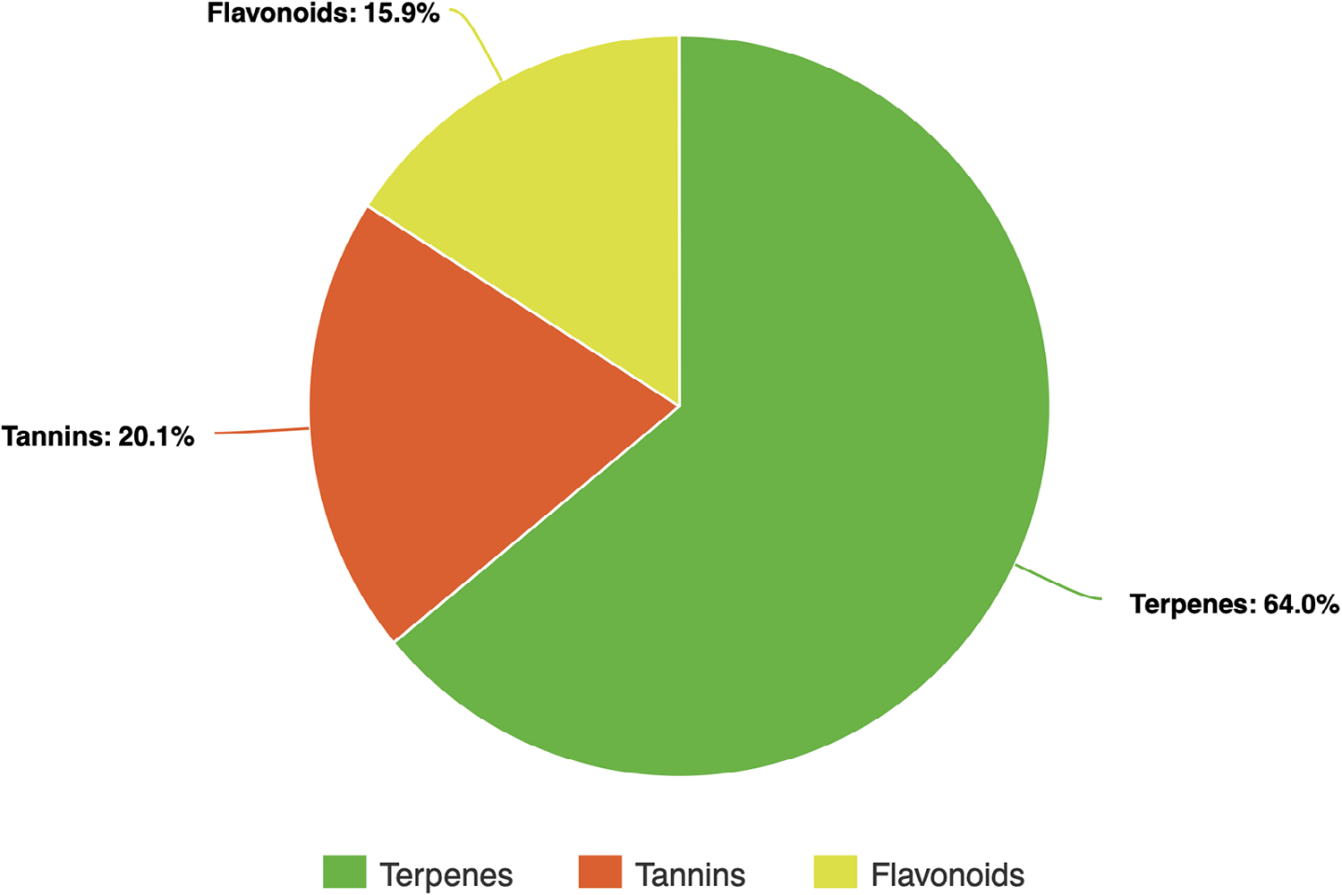
Relative distribution of the phytochemical classes identified in *P. pinea* periderm extract by UPLC–QTOF–MS operating in positive electrospray ionization (ESI+) mode. Percentages represent the relative contribution of each phytochemical class to the total identified compounds, calculated from the quantified amounts expressed as mg/g of dry extract: terpenes, 3.22 mg/g; tannins, 1.01 mg/g; flavonoids, 0.80 mg/g.

In positive electrospray ionization mode (ESI+), the total amount of annotated metabolites was 5.03 mg/g dry extract (Table 2). As shown in Figure 3, terpenoids were the predominant class (3.22 mg/g; 64.0%), followed by condensed tannins (1.01 mg/g; 20.1%) and flavonoids (0.80 mg/g; 15.9%). The most abundant metabolites detected in ESI(+) included procyanidin B C‐glucoside isomer 2 (1.01 ± 0.01 mg/g), pubescenol (1.01 ± 0.04 mg/g), 24,25‐diacetylbulgaroside (0.82 ± 0.03 mg/g), and delphinidin (0.80 ± 0.05 mg/g).

**TABLE 2:** Compounds identified in *P. pinea* periderm extract by UPLC–QTOF–MS in ESI(+) mode.

RT (min)	*m/z*	Molecular formula [M+H]^+^	Main fragments	Name	Class	mg/g extract
4.68	741.2005	C_36_H_36_O_17_	723.1925, 467.1199	Procyanidin B, C‐glucoside isomer 2	Tannins	1.01 ± 0.01
6.41	331.1552	C_19_H_22_O_5_	—	Gibberellin A7	Terpenes	0.12 ± 0.01
6.60	303.0524	C_15_H_11_O_7_	285.0399, 275.0548	Delphinidin	Flavonoids	0.80 ± 0.05
9.38	369.2647	C_21_H_36_O_5_	—	gamma‐Eudesmol rhamnoside	Terpenes	0.24 ± 0.03
11.71	485.2892	C_29_H_44_O_8_	443.2790	24,25‐Diacetylvulgaroside (M+H–2H_2_O)	Terpenes	0.82 ± 0.03
11.90	475.3042	C_28_H_42_O_6_	457.2941	Pubescenol	Terpenes	1.01 ± 0.04
14.61	338.3424	C_20_H_40_O	—	Phytol (M+ACN+H)	Terpenes	0.50 ± 0.06
18.13	413.3773	C_29_H_48_O	—	Stigmasterol	Terpenes	0.53 ± 0.13

Overall, the complementary acquisition in both ionization modes highlighted the chemical complexity of the *P. pinea* periderm extract. While ESI(−) was particularly effective for the detection of polyphenolic constituents, especially condensed tannins and flavonoids, ESI(+) improved the characterization of terpenoid metabolites, providing a broader overview of the extract phytochemical composition.

### 3.3 Elemental Composition (TXRF Analysis)

**FIGURE 4:**
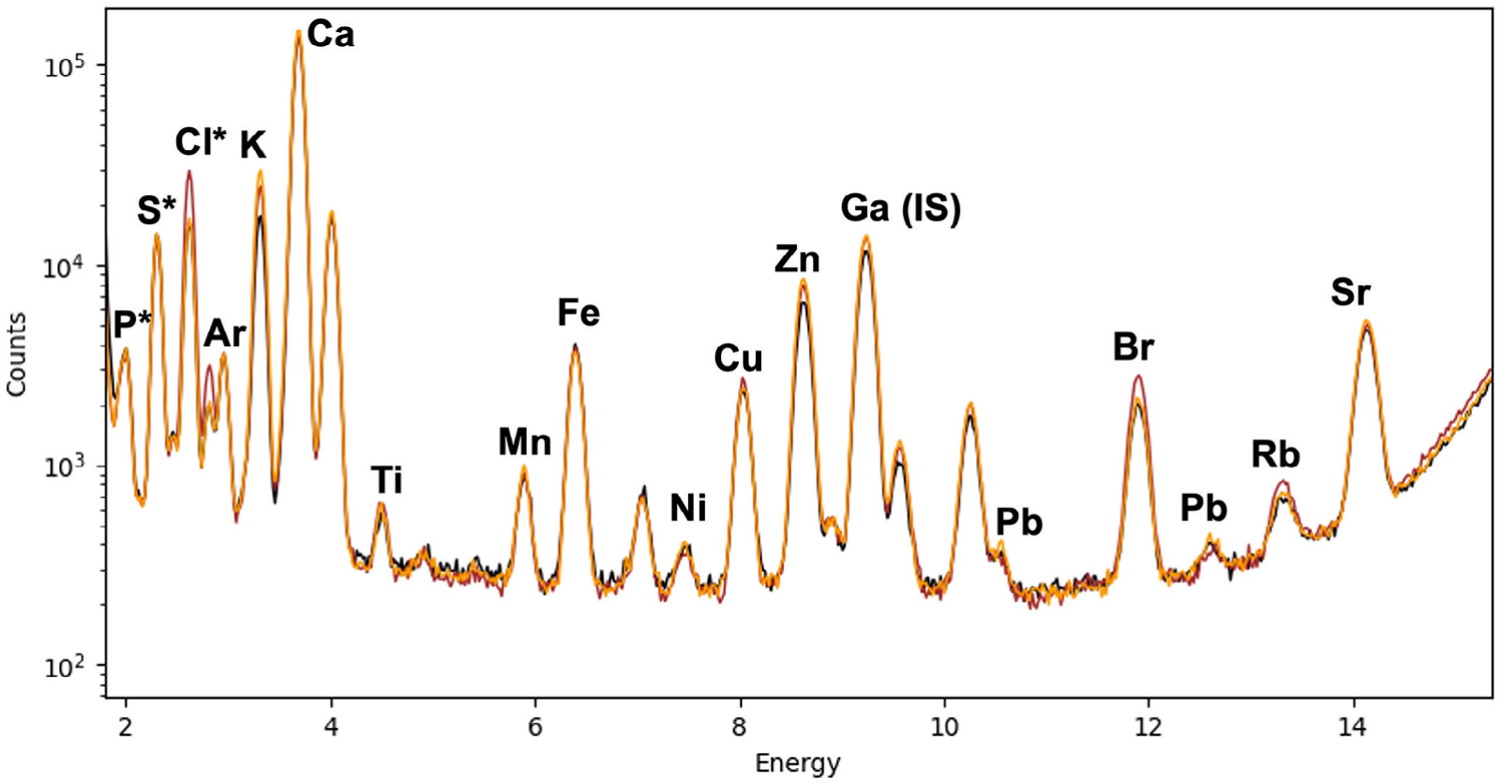
TXRF spectra of three replicates from the lyophilized hydroalcoholic extract obtained from *P. pinea* periderm prepared as a suspension. Ga peak corresponds to the internal standard added to perform the quantitative analysis.

Figure 4 presents the TXRF spectra of three replicates of the lyophilized hydroalcoholic extract of *P. pinea*, prepared as a suspension and irradiated for 600 s. The qualitative analysis of TXRF measurements identifies the presence of P, S, Cl, K, Ca, Ti, Mn, Fe, Ni, Cu, Zn, Pb, Br, Rb, and Sr, while Ga is the internal standard added for quantification of these elements. Quantitative analysis was performed starting from K because significant absorption effects occur for light elements such as P, S, and Cl, causing signal attenuation and potential underestimation of their concentrations under air measurement conditions.

The results of the quantitative analysis are summarized in Table 3. TXRF analysis revealed the presence of essential mineral nutrients such as K (0.095%) and Ca (0.7%), which accounted for the majority of the extract's inorganic content, consistent with the typical mineral composition reported for plant‐derived matrices [36, 37]. Fe, Cu, Zn, and Sr were detected as minor elements, with concentrations ranging from 18 to 50 mg/kg, whereas Mn, Ni, Br, and Rb were present at trace levels (<10 mg/kg). Although Pb was detected at low levels (1.40 mg/kg), its concentration remained below the maximum limits established for contaminants in food according to the Codex Alimentarius standards (FAO/WHO) [38]. Ti was also detected at trace levels (<10 mg/kg); however, titanium is not generally considered a regulated food contaminant, and its presence in plant‐derived materials is commonly attributed to soil‐derived particles or environmental deposition on plant tissues.

**TABLE 3:** Concentration of major, minor, and trace elements obtained by TXRF analysis of lyophilized extract of *P. pinea* periderm.

Element	Mean (ppm)	±	S.D
**K**	950	±	250
**Ca**	7000	±	400
**Ti**	7.0	±	1.5
**Mn**	9.4	±	0.7
**Fe**	45	±	12
**Ni**	1.0	±	0.4
**Cu**	18	±	2
**Zn**	46	±	9
**Pb**	1.4	±	0.4
**Br**	10	±	2
**Rb**	0.38	±	0.15
**Sr**	25	±	2

*Note*: Values are presented as mean ± SD (*n* = 3 independent measurements).

### 3.4 DPPH Radical‐Scavenging Activity

The DPPH assay showed that *P. pinea* periderm extract possesses marked radical‐scavenging activity (Table 4). As described in the Methods section, extract concentrations were expressed as dry lyophilized extract equivalents, while taxifolin was tested at concentrations corresponding to the estimated amount of taxifolin present in each extract dilution.

**TABLE 4:** DPPH radical‐scavenging activity of *P. pinea* periderm extract and taxifolin standard.

Sample	Concentration (µg/mL)	Scavenging activity (%)	Trolox equivalents (µg/mL)	Ascorbic acid equivalents (µg/mL)
*P. pinea* periderm extract	2.5	34.65 ± 1.29	5.14	6.46
	5	41.51 ± 1.90	6.15	7.74
	25	79.63 ± 2.07	11.81	14.85
	50	87.34 ± 2.44	12.95	16.29
Taxifolin	0.05	23.66 ± 0.36	3.51	4.41
	0.1	33.93 ± 5.31	5.03	6.33
	0.5	35.01 ± 3.41	5.19	6.53
	1.0	39.90 ± 1.43	5.92	7.44

*Note*: Data are expressed as percentage of DPPH radical scavenging and reported as mean ± SD (*n* = 5). Concentrations of *P. pinea* periderm extract are expressed as dry lyophilized extract equivalents. Taxifolin concentrations correspond to the amount of taxifolin estimated by HPLC–DAD to be present in the respective extract concentrations reported above. Trolox equivalent and ascorbic acid equivalent values were calculated using the linear regression equations of the corresponding reference standards.

The extract showed a marked concentration‐dependent radical‐scavenging activity, increasing from 34.65% ± 1.29% at 2.5 µg/mL to 87.34% ± 2.44% at 50 µg/mL. The antioxidant capacity expressed as Trolox equivalent and ascorbic acid equivalent values followed the same concentration‐dependent trend (Table 4). Under the same taxifolin‐equivalent comparison, pure taxifolin produced lower scavenging values, ranging from 23.66% ± 0.36% at 0.05 µg/mL to 39.90% ± 1.43% at 1.0 µg/mL.

This comparison indicates that the amount of taxifolin present in the extract accounts for only part of the radical‐scavenging activity observed for the whole extract. For instance, the extract at 50 µg/mL produced 87.34% ± 2.44% inhibition, whereas the corresponding concentration of pure taxifolin (1.0 µg/mL) produced 39.90% ± 1.43% inhibition. Therefore, the antioxidant response of the extract is likely supported by the broader phytochemical matrix rather than by taxifolin alone.

Dose–response analysis further confirmed the strong antioxidant activity of the extract, as summarized by the IC_50_ values reported in Table 5. The IC_50_ value was 12.31 µg/mL when expressed as dry lyophilized extract, compared with 14.57 µg/mL for pure taxifolin. The reference antioxidants Trolox and ascorbic acid showed IC_50_ values of 6.72 and 13.94 µg/mL, respectively. Overall, the extract displayed DPPH radical‐scavenging potency comparable to that of ascorbic acid and taxifolin, while the direct taxifolin‐equivalent comparison supports a relevant contribution from additional extract constituents.

**TABLE 5:** DPPH radical‐scavenging activity expressed as IC_50_ values for *P. pinea* periderm extract, taxifolin, ascorbic acid, and Trolox.

Sample	IC_50_ (µg/mL)
*P. pinea* periderm extract	12.31
Taxifolin	14.57
Ascorbic acid	13.94
Trolox	6.72

### 3.5 In Vitro Cytocompatibility Assessment

**FIGURE 5:**
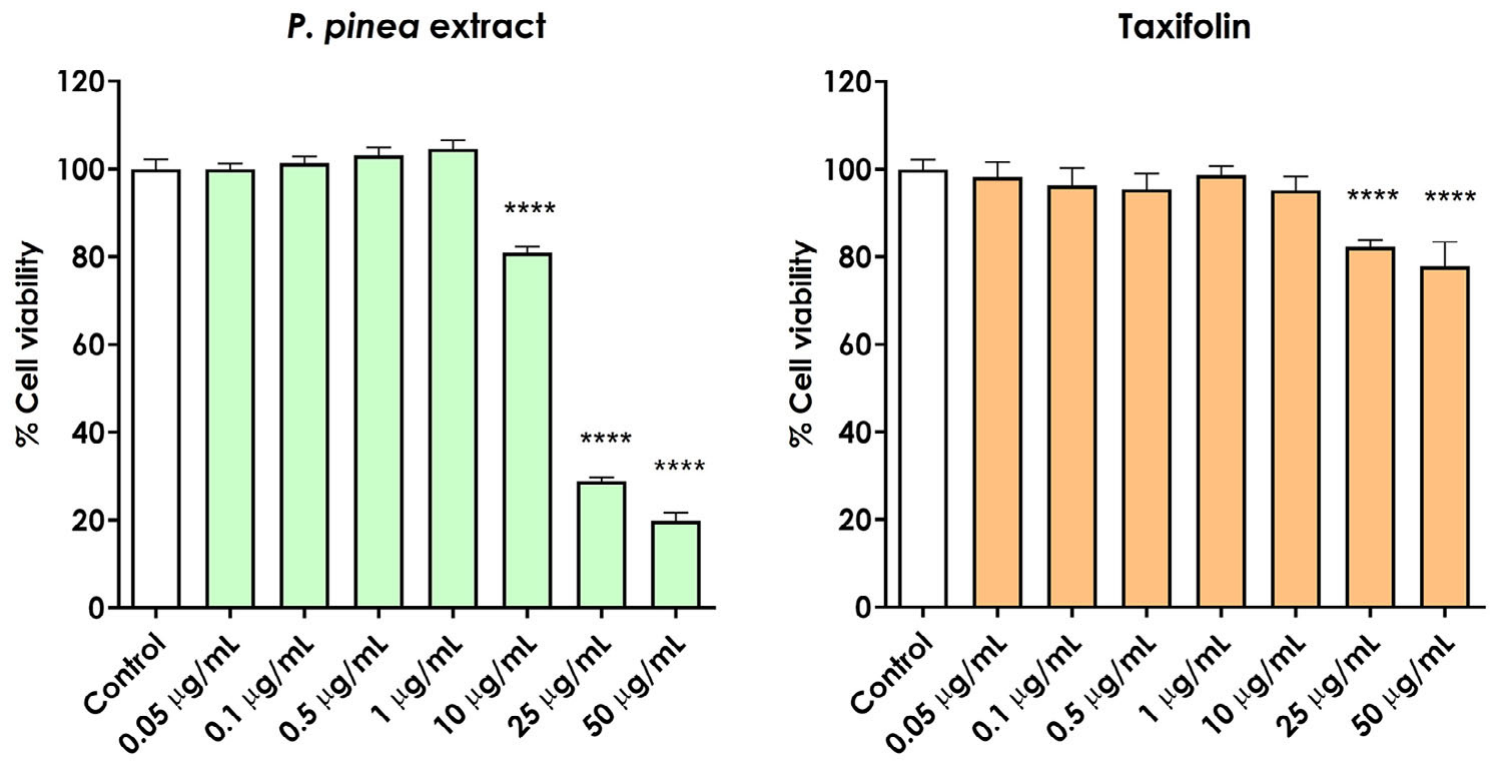
Cytocompatibility of *P. pinea* periderm extract and taxifolin in SH‐SY5Y cells assessed by the MTT assay. SH‐SY5Y cells were treated for 24 h with increasing concentrations of *P. pinea* periderm extract (left panel) or taxifolin (right panel). Cell viability is expressed as a percentage relative to untreated control cells. Concentrations of the extract are expressed according to its taxifolin (TAX) content, as quantified by HPLC–DAD analysis. Data are presented as mean ± SEM from three independent biological experiments (*n* = 3), each performed with three technical replicates per condition. Statistical analysis was performed using one‐way ANOVA followed by Dunnett's two‐sided multiple‐comparisons test versus the untreated control. ****p ≤ 0.0001.

The cytotoxicity of the *P. pinea* periderm extract was evaluated in SH‐SY5Y cells using the MTT assay after 24 h of exposure to increasing concentrations ranging from 0.05 to 50 µg/mL (Figure 5).

At concentrations between 0.05 and 1 µg/mL, the extract did not adversely affect cell viability, which remained comparable to or slightly above that of untreated control cells. A modest reduction in viability was observed at 10 µg/mL, where cell viability decreased to approximately 80% of control values. At higher concentrations (25 and 50 µg/mL), a marked decrease in cell viability was detected, with values ranging approximately from 30% to 20%, respectively. Taxifolin, tested under the same experimental conditions, showed a different cytocompatibility profile (Figure 5). Cell viability remained close to control levels up to 10 µg/mL, while a significant reduction was observed at 25 and 50 µg/mL, where viability dropped below the 80% threshold, consistent with its well‐documented anticancer activity.

Overall, these results identify a concentration range up to 1 µg/mL for the *P. pinea* periderm extract in which no cytotoxic effects were observed, providing the basis for the selection of non‐cytotoxic concentrations for subsequent oxidative stress protection assays.

### 3.6 In Vitro Antioxidant Activity Assessment

**FIGURE 6:**
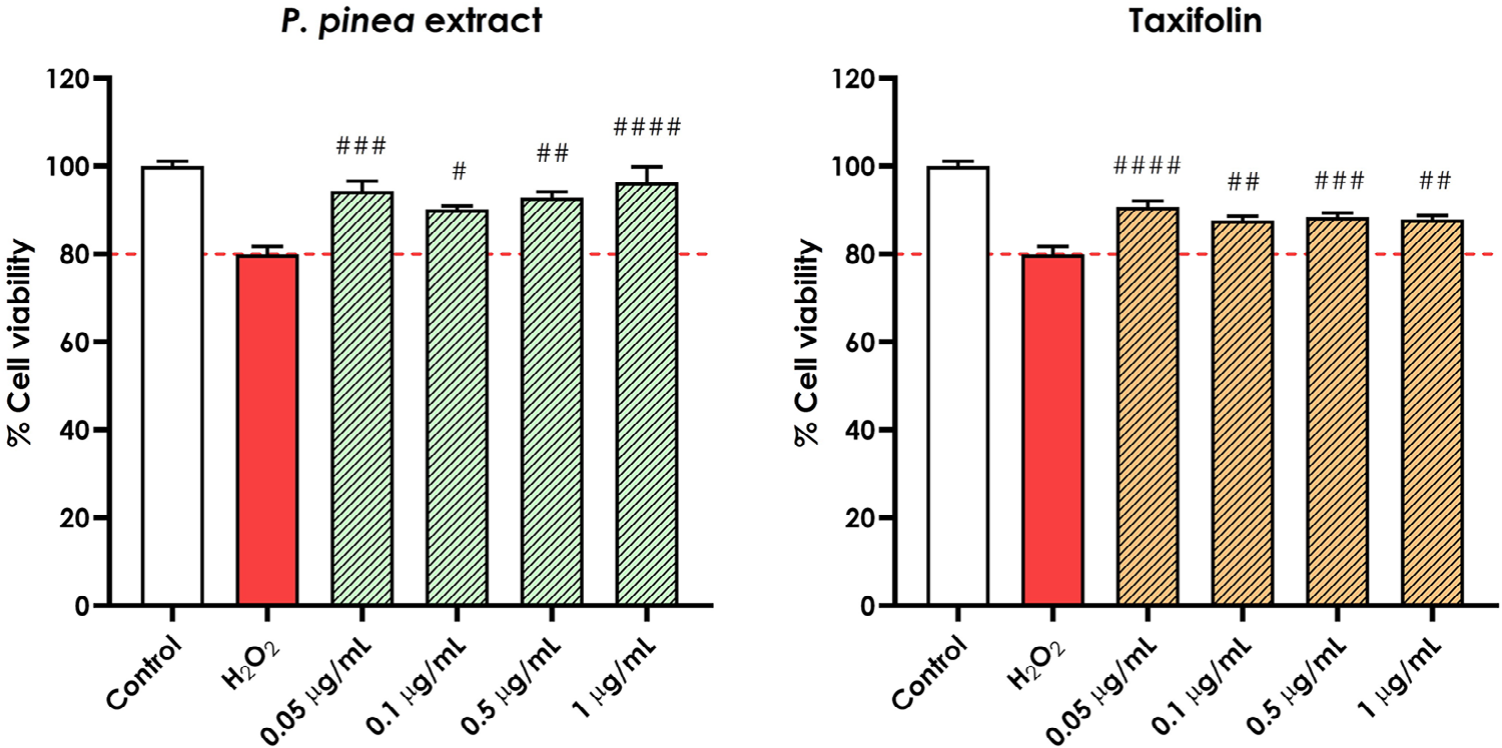
Protective effects of *P. pinea* periderm extract and taxifolin against H_2_O_2_‐induced oxidative stress in SH‐SY5Y cells. SH‐SY5Y cells were pre‐treated for 24 h with increasing concentrations of *P. pinea* periderm extract (left panel) or taxifolin (right panel), followed by H_2_O_2_ exposure (100 µM, 2 h). Cell viability was assessed by the MTT assay and expressed as a percentage relative to untreated control cells. The dashed red line represents the 80% viability threshold commonly used to define non‐cytotoxic conditions. Data are presented as mean ± SEM from three independent biological experiments (*n* = 3), each performed with three technical replicates per condition. Statistical analysis was performed using one‐way ANOVA followed by Dunnett's two‐sided multiple‐comparisons test versus the H_2_O_2_‐treated group. ^#^ *p* ≤ 0.05; ^##^ *p* ≤ 0.01; ^###^ *p* ≤ 0.001; ^####^ *p* ≤ 0.0001.

The protective effects of the *P. pinea* periderm extract against oxidative stress were evaluated in SH‐SY5Y cells using an H_2_O_2_‐induced cytotoxicity model. Exposure to H_2_O_2_ (100 µM, 2 h) reduced cell viability to approximately 80% compared to untreated control cells (Figure 6). Pre‐treatment with the *P. pinea* periderm extract for 24 h partially counteracted the reduction in cell viability induced by H_2_O_2_ at all non‐cytotoxic concentrations tested (0.05–1 µg/mL), which were selected based on the cytocompatibility assay. At these concentrations, cell viability was restored to values ranging between approximately 90% and 100%, with statistically significant differences compared to H_2_O_2_‐treated cells. Similarly, taxifolin pre‐treatment improved cell viability after H_2_O_2_ exposure at concentrations between 0.05 and 1 µg/mL, restoring viability to levels above the 80% threshold.

Overall, both the *P. pinea* periderm extract and taxifolin improved cell viability after H_2_O_2_ exposure within a comparable non‐cytotoxic concentration range, suggesting a preliminary cytoprotective effect under oxidative stress conditions. Since this assay was based on MTT‐derived cell viability, further studies measuring intracellular ROS levels, apoptosis markers and mitochondrial function will be required to clarify the underlying mechanisms.

## 4 Discussion

The present study investigates *P. pinea* periderm through an integrated approach combining phytochemical profiling, antioxidant evaluation, cell‐based assays and elemental analysis. Although *P. pinea* is one of the most widespread and economically important pine species in the Mediterranean region, its bark‐derived tissues, particularly the periderm, remain poorly characterized compared with those of other members of the genus *Pinus*.

From a phytochemical perspective, the UPLC–QTOF–MS profile of *P. pinea* periderm shows several similarities with bark extracts from other Pinus species, particularly *P. pinaster* [10, 11] and *P. radiata* [39, 40]. These species are known to contain flavonoids and condensed tannins, compounds widely associated with the antioxidant properties of pine bark extracts, including Pycnogenol [41, 42]. The detection of similar phytochemical classes in *P. pinea* periderm suggests that this underexplored tissue shares relevant compositional features with better‐characterized pine bark matrices, while also highlighting its potential as a renewable source of phenolic secondary metabolites.

Among the annotated metabolites, taxifolin was the most abundant individual compound. This flavonoid has been widely reported in pine bark extracts, including those from *P. pinaster* [41], *P. sylvestris* and *P. radiata* [14], and is commonly associated with antioxidant and anti‐inflammatory properties. Its abundance in *P. pinea* periderm supports its use as a representative marker compound for this extract. However, the DPPH assay indicated that taxifolin alone does not fully explain the antioxidant activity of the whole extract. Indeed, when tested at concentrations corresponding to those estimated in the extract, pure taxifolin produced lower radical‐scavenging values than the complete phytochemical matrix, suggesting the contribution of additional phenolic constituents, including condensed tannins and other flavonoids.

This observation is consistent with a growing body of evidence indicating that the antioxidant properties of plant extracts are often driven by the combined action of multiple phenolic constituents rather than by a single dominant compound [43, 44].

In particular, condensed tannins, catechins, epicatechin, and other flavonoid derivatives identified in the extract are known to exhibit strong antioxidant activity, especially when present within a phytocomplex, due to potential synergistic interactions. Several studies have demonstrated that oligomeric and polymeric proanthocyanidins display enhanced radical‐scavenging capacity compared to their monomeric counterparts, and that their activity is strongly influenced by structural features such as degree of polymerization and interflavan linkages [45, 46, 47]. Moreover, experimental evidence has shown that multicomponent flavonoid systems can exert additive or synergistic antioxidant effects, resulting in a higher overall activity than that predicted from individual constituents alone [44, 48].

Similar behavior has been reported for pine bark extracts, including *P. pinaster*‐derived preparations, where antioxidant effects are generally attributed to the combined contribution of flavonoids and proanthocyanidins rather than to isolated compounds [42, 49]. In this context, the DPPH activity observed for the *P. pinea* periderm extract is likely related to the overall phytochemical composition of the extract, particularly the presence of condensed tannins, flavonoids and other phenolic constituents. This supports the interpretation of the extract as a complex phytochemical matrix rather than as a source of a single antioxidant compound.

The biological relevance of the complex phytochemical profile observed in *P. pinea* periderm was further supported by cellular assays in the SH‐SY5Y neuroblastoma model, a widely used in vitro cellular model for studying oxidative stress‐mediated neuronal damage and protection [24, 50].

Consistent with this model, several natural phenolic‐rich extracts and isolated flavonoids have been reported to attenuate H_2_O_2_‐induced cytotoxicity and reduce ROS production in SH‐SY5Y cells, often through modulation of antioxidant defenses and apoptosis‐related pathways [51, 52, 53]. For example, flavan‐3‐ols such as catechin have been widely reported to exert neuroprotective effects against oxidative stress through their ability to modulate intracellular redox balance and cellular signaling pathways involved in neuronal survival [51]. Similarly, flavonoids such as quercetin have been shown to enhance neuronal resistance to oxidative stress through the regulation of antioxidant defense mechanisms [52]. In addition, taxifolin (dihydroquercetin), a major constituent identified in the present extract, has been reported to exert cytoprotective effects in neuronal models through its antioxidant and mitochondrial protective properties [53].

Notably, mixtures of phenolic antioxidants have been reported to exert additive or synergistic effects in oxidative stress models [54]. In the present study, both *P. pinea* periderm extract and taxifolin improved SH‐SY5Y cell viability after H_2_O_2_ exposure within non‐cytotoxic concentration ranges. However, since this evaluation was based on MTT‐derived cell viability, these findings should be interpreted as preliminary evidence of a cytoprotective effect rather than as direct proof of a specific antioxidant mechanism. Further studies measuring intracellular ROS levels, apoptosis markers, and mitochondrial function will be required to clarify the mechanisms underlying the observed response.

Beyond chemical and biological considerations, the use of periderm as the source material confers additional ecological and sustainability value. Periderm is a secondary protective tissue that constitutes the outermost barrier of bark in woody plants and is formed during secondary growth [55]. Unlike invasive debarking practices, known to affect bark regeneration and, in severe cases, tree survival, the collection of naturally renewed outer bark/periderm can be framed as a low‐impact approach when performed superficially [56]. In this perspective, periderm valorization aligns with circular bioeconomy concepts and sustainable forestry by enabling the conversion of low‐value bark side‐streams into sources of high‐value bioactive compounds [57, 58].

An additional strength of the present study is the inclusion of elemental analysis as part of the overall characterization of the *P. pinea* periderm extract. Pine bark‐derived products are already established in the nutraceutical field, with Pycnogenol, a standardized extract obtained from *P. pinaster* bark, representing one of the most widely used references. Pycnogenol is commonly administered at a recommended daily dose of approximately 100 mg of dry extract [42], providing a practical benchmark for contextualizing elemental exposure from pine‐derived botanical preparations.

Assuming a comparable daily intake for the *P. pinea* periderm extract, the concentrations of regulated heavy and trace elements quantified by TXRF correspond to estimated daily intakes that remain well below the permitted daily exposure limits established for oral administration [59, 60, 61]. In particular, elements of major toxicological concern were detected only at low concentrations, resulting in estimated exposure levels markedly lower than the conservative limits established by international safety guidelines for elemental impurities, including the ICH Q3D (R5) guideline for pharmaceuticals [62] and the Codex Alimentarius Commission issued by FAO and WHO for food and botanical products [38].

This aspect is especially relevant considering increasing reports of metal contamination in botanical supplements [50, 63, 64], where chronic exposure may pose significant health risks despite the absence of acute cytotoxic effects in vitro. The TXRF data indicate that, under the assumed supplementation scenario, the elemental profile of the *P. pinea* periderm extract remains within conservative safety margins for regulated elements. However, this assessment should be considered preliminary, since actual exposure, bioavailability, absorption, and long‐term accumulation of metals were not evaluated in the present study. Therefore, while the elemental profile provides a favorable safety‐oriented indication, further toxicological and bioavailability studies will be required before drawing definitive conclusions regarding nutraceutical suitability.

Taken together, the present findings provide a robust initial characterization of *P. pinea* periderm as a source of natural bioactive compounds. Although the study is based on in vitro data, the integrated phytochemical, antioxidant, cellular, and elemental evidence highlights this previously unexplored tissue as a promising matrix for further investigation within the field of plant‐derived antioxidant sources. Future studies should expand upon these results by including a broader range of in vitro systems, encompassing both immortalized cell lines and primary cells of different tissue origin, to better define the biological relevance and reproducibility of the observed effects. Mechanistic investigations focusing on redox regulation, such as intracellular ROS modulation, mitochondrial function and the regulation of endogenous antioxidant defense pathways, will be essential to clarify the molecular basis of the cytoprotective activity. Furthermore, in vivo studies will be required to evaluate bioavailability, metabolism and long‐term safety, thereby defining the nutraceutical and functional potential of *P. pinea* periderm extracts.

## 5 Conclusion

This study shows that *P. pinea* periderm is a promising renewable bark‐derived matrix rich in secondary metabolites, particularly condensed tannins and flavonoids, with taxifolin as the main quantified marker compound. The hydroalcoholic extract displayed relevant DPPH radical‐scavenging activity and improved SH‐SY5Y cell viability under H_2_O_2_‐induced oxidative stress within non‐cytotoxic concentrations, although these effects should be considered preliminary and not yet mechanistically defined. The comparison with taxifolin indicates that the antioxidant response of the extract is likely supported by the combined contribution of multiple phenolic constituents rather than by a single compound. TXRF analysis further provided a preliminary safety‐oriented elemental profile, showing low levels of regulated elements under the assumptions considered. Overall, these findings support the potential valorization of *P. pinea* periderm as a sustainable source of antioxidant secondary metabolites. Further studies addressing intracellular oxidative stress markers, bioavailability, long‐term safety, and in vivo relevance will be necessary to confirm its functional applicability.

## Author Contributions

**Vlad Sebastian Popescu**: conceptualization, methodology, investigation, formal analysis, data curation, visualization, validation, writing – original draft, and writing – review and editing; **Fabjola Bilo**: methodology, formal analysis, investigation, resources, and writing – review and editing; **Eileen Mac Sweneey**: investigation; **Laura Borgese**: resources, methodology, and supervision; **Gregorio Peron**: investigation, methodology, formal analysis, and writing – review and editing; **Giulia Abate**: investigation, resources, and writing – review and editing; **Andrea Mastinu**: supervision, project administration, and writing – review and editing.

## Conflicts of Interest

The authors declare no conflicts of interest.

## Declaration of Generative AI and AI‐Assisted Technologies in the Writing Process

During the preparation of this manuscript, the authors used ChatGPT (OpenAI) to assist with language editing and text refinement. The authors carefully reviewed and edited the generated content and take full responsibility for the final version of the manuscript.

## Data Availability

The data supporting the findings of this study are available from the corresponding author upon reasonable request.
